# Association of serum galectin-3 levels with mortality and cardiovascular disease outcomes in hemodialysis patients: a systematic review and dose–response meta-analysis

**DOI:** 10.1007/s11255-024-04026-4

**Published:** 2024-03-22

**Authors:** Ioannis Bellos, Smaragdi Marinaki, Pagona Lagiou, Vassiliki Benetou

**Affiliations:** 1 Department of Hygiene, Epidemiology and Medical Statistics, National and Kapodistrian University of Athens, Medical School, 75, Mikras Asias Str., 115 27 Athens, Greece; 2 Department of Nephrology and Renal Transplantation, Laiko General Hospital, National and Kapodistrian University of Athens, Medical School, 75, Mikras Asias Str., 115 27 Athens, Greece

**Keywords:** Galectin, Dialysis, Kidney failure, Mortality, Cardiovascular, Meta-analysis

## Abstract

**Background:**

Galectin-3 has been proposed as a candidate marker for cardiovascular risk stratification, although its role in kidney failure is unclear. The aim of this systematic review was to assess the association of serum galectin-3 levels with overall survival and cardiovascular outcomes among hemodialysis patients.

**Methods:**

Medline, Scopus, Web of Science and CENTRAL were systematically searched from inception till Aug 20, 2023. Observational studies evaluating the association of serum galectin-3 with mortality, cardiovascular disease and arterial stiffness in hemodialysis patients were included. The exposure–response relationship between galectin-3 and mortality was explored by dose–response meta-analysis using restricted cubic splines in a one-stage approach.

**Results:**

Overall, 13 studies were included (9 cohort and 4 cross-sectional), comprising 6025 hemodialysis individuals. Increasing galectin-3 values were associated with greater all-cause mortality risk (*χ*^*2*^: 18.71, *p-value* < 0.001) and an insignificant trend toward higher cardiovascular mortality risk (*χ*^*2*^: 5.06, *p-value*: 0.079). Compared to a reference galectin-3 value of 10 ng/ml, all-cause mortality risk was significantly higher with levels of 20 ng/ml (Hazard ratio–HR: 2.62, 95% confidence intervals-CI: 1.66–4.15), 30 ng/ml (HR: 3.78, 95% CI: 2.05–6.97) and 40 ng/ml (HR: 4.01, 95% CI: 2.14–7.52). Qualitative synthesis of evidence indicated that serum galectin-3 may be linked to abdominal aortic calcification severity and progression, as well as to left ventricular systolic and diastolic dysfunction.

**Conclusions:**

This study suggests that high serum galectin-3 levels are associated with greater all-cause mortality risk among patients on maintenance hemodialysis. Preliminary cross-sectional evidence indicates that serum galectin-3 may be associated with arterial stiffness and left ventricular dysfunction.

## Introduction

End-stage kidney disease affects more than 4 million people worldwide, with hemodialysis representing the main form of kidney replacement therapy [1]. Maintenance hemodialysis is associated with high direct and indirect economic costs [2], as well as with poor quality of life, significant morbidity and 5-year survival rates below 50% despite significant improvements in dialysis practices [3, 4]. Cardiovascular disease affects the majority of hemodialysis patients due to the high prevalence of traditional risk factors, especially hypertension and diabetes mellitus, in conjunction with non-traditional factors, such as metabolic acidosis and accumulation of uremic toxins [5]. Cardiovascular disease may present in different clinical forms, varying from myocardial infarction and stroke to heart failure and fatal arrhythmias. Importantly, sudden cardiac arrest constitutes the most common cause of cardiovascular death in hemodialysis patients, reflecting the effects of intradialytic hypotension and electrolyte imbalances [6]. As a result, growing research interest exists in the field of novel biomarker evaluation in order to promote effective cardiovascular risk stratification of hemodialysis patients, enabling the identification of individuals that would benefit the most from intensive follow-up and early intervention [7].

Galectin-3 is a *β*-galactoside-binding lectin, which is secreted by endothelial cells, vascular smooth muscle cells and macrophages [8]. Based on the organization of their carbohydrate-recognition domains, galectins are categorized in the proto-type group, the tandem-repeat group and the chimera-type group which includes only galectin-3 [9]. At the cellular level, galectins are synthesized in free cytoplasmic polysomes and are subsequently secreted after interacting with glycan ligands [10]. Intracellular galectins are involved in various physiologic and pathophysiologic pathways, including apoptosis and mRNA splicing [11]. Galectins also exert a variety of actions at the cellular surface and is implicated in inflammation and innate immunity, functioning as pattern recognition receptors or as effector molecules [12].

More specifically, galectin-3 serves as a regulator of inflammatory responses, contributing to the regulation, cell proliferation and apoptosis, as well as to extracellular matrix deposition and the development of fibrosis [13]. High serum galectin-3 has been associated with heart failure incidence and adverse outcomes [14, 15]; hence, the 2017 ACC/AHA/HFSA (American College of Cardiology/American Heart Association/Heart Failure Society of America) have incorporated the biomarker as a possibly useful tool for risk stratification of chronic heart failure patients [16]. In addition, galectin-3 has been suggested to be implicated in the pathophysiology of atherosclerosis and vascular calcification [17], presenting potentially prognostic value in patients with myocardial infarction [18] and acute stroke [19].

Galectin-3 has been proposed to contribute to the pathogenetic processes of both acute kidney injury and chronic kidney disease [20], while its serum levels present an inverse association with kidney function [21]. Its high values have been shown to increase the risk of diabetic nephropathy [22], as well as to predict rapid progression [23] and adverse outcomes in patients with pre-dialysis chronic kidney disease [24]. The aim of the present systematic review is to accumulate the existing literature knowledge in the field and assess the association of serum galectin-3 levels with overall survival and cardiovascular outcomes among hemodialysis individuals.

## Materials and methods

### Study design

The present systematic review and meta-analysis was reported following the Preferred Reporting Items for Systematic Reviews and Meta-Analysis (PRISMA) guidelines [25]. The protocol of the study has been prospectively registered and is publicly available (dx.doi.org/10.17504/protocols.io.j8nlkooxxv5r/v1). No ethical approval was required as no new participants were recruited and already published data were used.

### Eligibility criteria

The population of the study consisted of adult patients with end-stage kidney disease undergoing maintenance hemodialysis. Pre-dialysis and peritoneal dialysis patients, as well as kidney transplant recipients, were excluded. The exposure of interest was serum galectin-3 levels. The primary endpoint was all-cause mortality, while secondary outcomes of interest included cardiovascular mortality, major adverse cardiovascular events, arterial stiffness and echocardiographic indices. Cohort (prospective and retrospective), case–control and cross-sectional studies were held eligible. Descriptive, animal and in vitro studies, as well as case reports/series, conference abstracts and review articles, were excluded.

### Literature search

The primary search was conducted by systematically searching from inception the following databases: Medline (via PubMed), Scopus, Web of Science and Cochrane Central Register of Controlled Trials (CENTRAL). Secondarily, Google Scholar, as well as the full reference lists of the included studies (“*snowball*” method [26]), were also searched, aiming to identify potentially missing articles. The date of the last search was set at Aug 20, 2023. Both MeSH (Medical Subject Headings) terms and keywords were used. The main search algorithm was the following: (“Renal Dialysis” [Mesh] OR “Kidney Failure, Chronic” [Mesh] OR dialysis OR hemodialysis OR “end-stage renal disease” OR “end-stage kidney disease” OR ESKD OR ESRD OR “kidney failure” OR “renal failure” OR “kidney insufficiency” OR “renal insufficiency”) AND (galectin OR “galectin-3”). No language restrictions were applied.

### Study selection

The process of study selection followed three stages. At first, the titles and abstracts of all electronic records were screened for potential eligibility. Subsequently, all articles that were presumed to meet the inclusion criteria were retrieved in full-text form. Then, any studies that met any of the exclusion criteria were excluded from the review. The selection of studies was performed independently by two authors, resolving any discrepancies through their consensus.

### Data extraction

The following information was extracted from the included studies using pre-piloted forms: year of publication, country, eligibility criteria, sample size, study design, galectin-3 assay, participants’ age, sex, percentage of hypertension, diabetes mellitus, dialysis vintage and single-pool urea Kt/V. The necessary data for the evaluation of outcomes of interest were also collected. Data extraction was conducted by two reviewers independently, resolving potential disagreements after discussion with a third author.

### Quality assessment

The risk of bias in the included studies was evaluated with the ROBINS-I tool [27], adjusted for exposure studies, taking into account the following domains: confounding, selection of participants, classification of exposures, departures from intended exposures, missing data, measurement of outcomes and selection of the reported results. The assessment of risk of bias was performed by two authors in an independent way and potential discrepancies were resolved through the consensus of all authors.

### Data analysis

Statistical analysis was conducted in R-3.6.5 (“*dosresmeta*” package [28]). Confidence intervals (CI) were set at 95%. Dose–response meta-analysis was performed to define the potential exposure–response relationship between serum galectin-3 levels and mortality risk. In particular, a non-linear model using restricted cubic splines was applied in a one-stage approach [29]. Restricted cubic splines were located at the 25th, 50th and 75th percentiles of the serum galectin-3 level distribution.

## Results

### Study selection

**Fig. 1:**
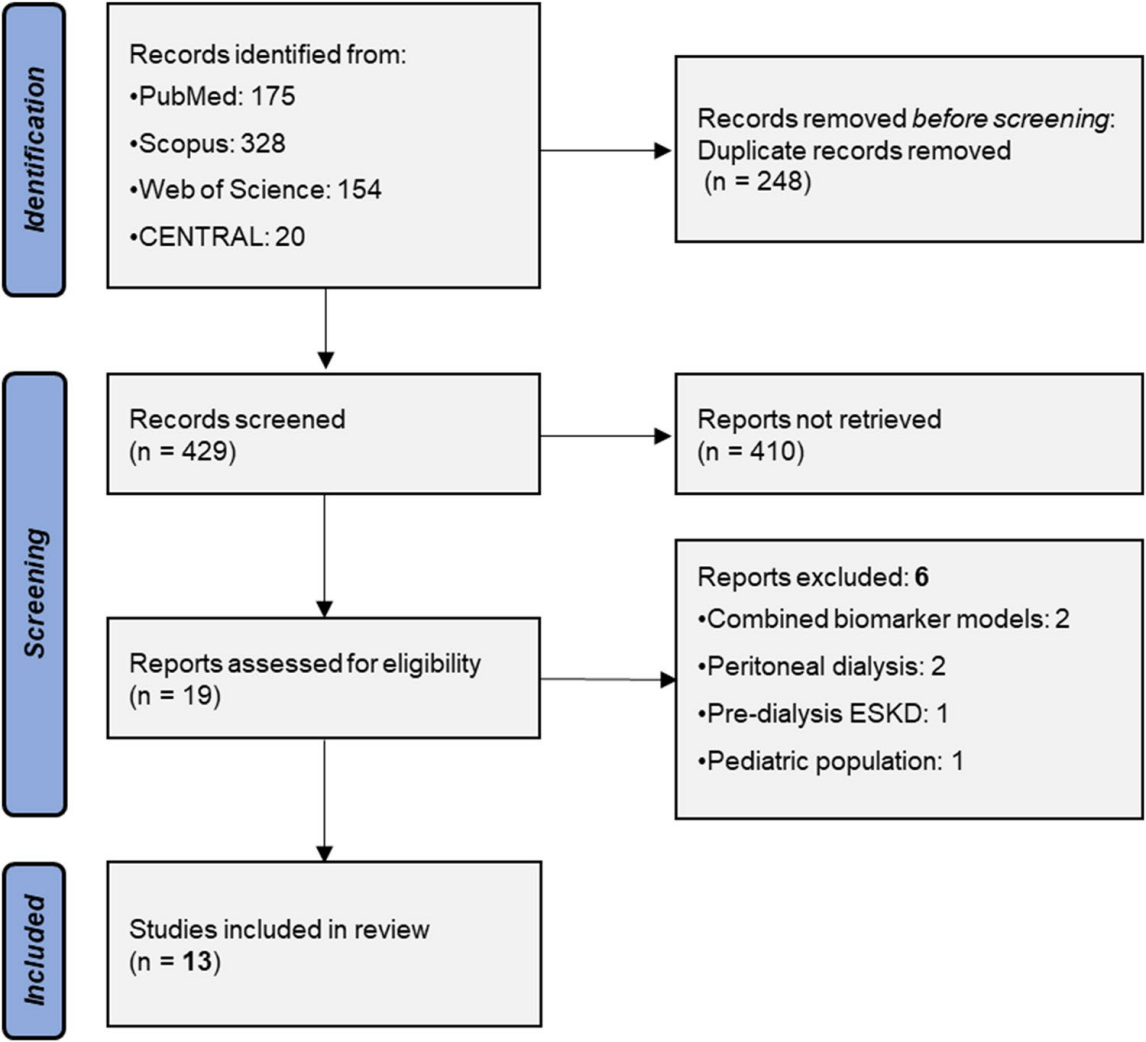
Search plot diagram. *ESKD* end-stage kidney disease

The process of study selection is schematically depicted in the PRISMA flowchart (Fig. 1). Overall, 677 electronic records were recognized through primary database search. After deduplication, 429 articles were screened for eligibility and 19 of them were retrieved as full texts. Then, six studies were excluded for evaluating galectin-3 only in combined models (*n* = 2) [30, 31], for including peritoneal dialysis (*n* = 2) [32, 33], pre-dialysis (*n* = 1) [34] or pediatric patients (*n* = 1) [35]. As a result, 13 studies [36–48] were included in the review, comprising a total of 6,025 hemodialysis patients.

### Included studies

The methodological characteristics of the included studies are presented in Table 1. A prospective cohort design was followed in nine studies, while four studies were cross-sectional ones. The majority of studies were conducted in Asia (ten studies), two studies took place in Europe and one study was international. The median follow-up period ranged from 22.2 to 60 months. The median age of participants was 60 years (interquartile range: 58.2 to 61), while 58% of patients were males. The median duration of dialysis was 58.2 months (interquartile range: 48.5–69.6). The outcomes of risk of bias assessment are presented in Table 2. Overall, 2 studies were evaluated to be at low risk of bias and 11 studies at moderate risk of bias. Risk of bias mainly arose from the domain of confounding due to the inadequate adjustment of potentially significant covariates, as well as from the domain of selection of the reported results since the lack of available protocols precluded the safe evaluation of whether subsets of estimates were omitted or selectively reported.

**Table 1:** Methodological and participant characteristics of the included studies

Study	Country	Study design	Sample size	Exclusion criteria	Gal-3 assay	Follow-up (months) ^†^	Age (years)^†^	Male sex (%)	Hypertension (%)	Diabetes mellitus (%)	Dialysis duration (months) ^†^	spKt/V^†^	Risk of bias
2022; Chou	Taiwan	Cross-sectional	176	Atrial fibrillation, below-the-knee amputation	Multiplex	NA	60	54	70.5	47.7	91.8	1.60	Moderate
2022; Liu	China	PC	506	Age ≥ 80 years, cancer	ELISA	60	58	53.4	NR	NR	51	1.37	Moderate
2022; Salib	Inter-national	PC	2343	Cancer, active infection, active liver disease, metabolic or gastrointestinal disease limiting life expectancy < 1 year	CMIA	45.6	64.2	62.1	NR	25.8	34.8	1.36	Low
2021; Kim	Korea	PC	296	Insufficient clinical data	ELISA	37.8	57	53	86.5	45.6	48.5	NR	Moderate
2020; Zhang	China	PC	284	Congestive heart failure, moderate/severe aortic stenosis, recent MI/stroke, cancer, acute infection	ELISA	31	61	58.1	83.8	12.7	90	1.60	Moderate
2020; Wang	China	PC	152	Age ≥ 85 years, liver failure, recent MI/stroke, active infection	ELISA	36	60	59.9	90	29.6	58.2	1.46	Moderate
2019; Zhang	China	Cross-sectional	311	Congestive heart failure, moderate/severe aortic stenosis, atrial fibrillation, recent MI/stroke, peripheral vascular disease, cancer, acute infection	ELISA	NA	61	57.2	78.5	20.6	90	1.67	Moderate
2018; Ko	Taiwan	PC	86	NYHA IV heart failure, cancer, acute infection, protein-energy wasting	ELISA	53.3	59.9	44.2	45.3	40.7	61.2	NR	Moderate
2016; Hogas	Romania	PC	88	NYHA IV heart failure, cancer, acute infection	ELISA	22.2	57.3	39.8	80.6	21.5	58.7	NR	Moderate
2016; Obokata	Japan	PC	423	Cancer	ELISA	25.2	66	68.8	84.6	46.6	69.6	1.4	Moderate
2015; Drechsler	Germany	PC	1168	No diabetes mellitus, active liver disease, hematopoietic disease, recent MI, resistant hypertension	ELISA	48	65.7	54.4	88.7	100	8.3	NR	Low
2015; Gurel	Turkey	Cross-sectional	87	Atrial fibrillation, LVEF < 50%, moderate/severe valvular disease, pericardial disease, uncontrolled hypertension	ELISA	NA	60.5	51.7	91	38	52.4	NR	Moderate
2015; Yilmaz	Turkey	Cross-sectional	105	Arrhythmia, moderate/severe valvular disease, diastolic-systolic dysfunction, liver disease, active infection	ELISA	NA	58.2	49.5	88.5	40.9	45	1.35	Moderate

**†**Median value

*NR* not reported, *NA* not applicable, *Gal-3* galectin-3, single-pool urea Kt/V, *PC* prospective cohort, *NYHA* New York Heart Association, *LVEF* left ventricle ejection fraction, *MI* myocardial infarction, *ELISA* enzyme-linked immunosorbent assay, *CMIA* chemiluminescent microparticle immunoassay

**Table 2:** Outcomes of the ROBINS-I evaluation

Study	Bias due to confounding	Bias in selection of participants into the study	Bias in classification of exposures	Bias due to deviations from intended exposures	Bias due to missing data	Bias in measurement of outcomes	Bias in selection of the reported result	Overall bias
2022; Chou	Low	Low	Low	Low	Low	Moderate	Low	Moderate
2022; Liu	Moderate	Low	Low	Low	NI	Low	Moderate	Moderate
2022; Salib	Low	Low	Low	Low	Low	Low	Low	Low
2021; Kim	Low	Low	Low	Low	Low	Low	Moderate	Moderate
2020; Zhang	Moderate	Low	Low	Low	NI	Low	Moderate	Moderate
2020; Wang	Moderate	Low	Low	Low	NI	Low	Low	Moderate
2019; Zhang	Moderate	Low	Low	Low	NI	Low	Low	Moderate
2018; Ko	Moderate	Low	Low	Low	NI	Low	Moderate	Moderate
2016; Hogas	Moderate	Low	Low	Low	NI	Low	Low	Moderate
2016; Obokata	Moderate	Low	Low	Low	Low	Low	Moderate	Moderate
2015; Gurel	Moderate	Low	Low	Low	Low	Low	Moderate	Moderate
2015; Drechsler	Low	Low	Low	Low	Low	Low	Low	Low
2015; Yilmaz	Moderate	Low	Low	Low	Low	Low	Moderate	Moderate

*NI* no information

### All-cause mortality

**Fig. 2:**
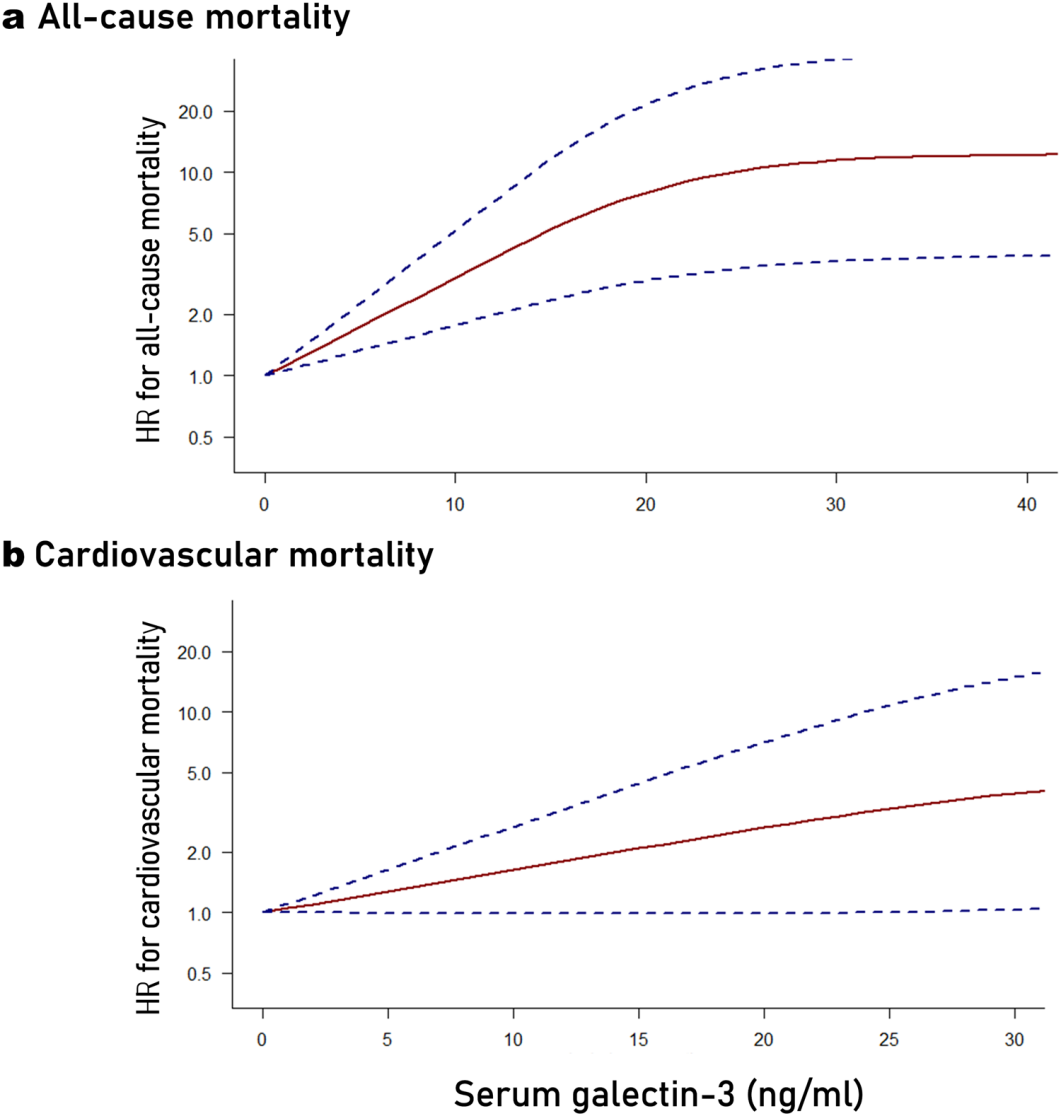
Relationship of serum galectin-3 levels with the risk of all-cause mortality (**a**) and cardiovascular mortality (**b**). Dashed lines indicate 95% confidence intervals

The outcomes of the qualitative synthesis of the included studies are described in Table 3. Galectin-3 was analyzed as a continuous variable in six prospective cohort studies; in four of them, increasing galectin-3 levels were associated with a significantly higher risk of all-cause mortality, while in two studies statistical significance was marginally not reached. The exposure–response relationship between serum galectin-3 and all-cause mortality is illustrated in Fig. 2a, showing that increasing galectin-3 values were associated with greater mortality risk (*χ*^*2*^: 18.71, *p-value* < 0.001). Compared to a reference serum galectin-3 value of 10 ng/ml, the risk of all-cause mortality was estimated to be significantly higher with galectin-3 levels of 20 ng/ml (Hazard ratio—HR: 2.62, 95% CI: 1.66–4.15), 30 ng/ml (HR: 3.78, 95% CI: 2.05–6.97) and 40 ng/ml (HR: 4.01, 95% CI: 2.14–7.52).

**Table 3:** Association of serum galectin-3 with mortality and cardiovascular outcomes

Study	All-cause mortality^†^	Cardiovascular mortality^†^	Death or MACE^†^	Arterial stiffness	Echocardiography
2022; Chou	–	–	–	*baPWV: β* = 0.1 (− 0.04; 0.20)	–
2022; Liu	*Per 1 Gal-3 unit:* 0.99 (0.96; 1.03) *Gal-3* > *8.65 ng/ml:* 1.59 (0.96; 2.65)	*Per 1 Gal-3 unit:* 1.01 (0.98; 1.05) *Gal-3* > *8.65 ng/ml:* 2.13 (1.07; 4.26) *			
2022; Salib	*Per 1 Gal-3 SD:* 1.18 (1.10–1.25)* *Gal-3: 56.3–78 ng/ml:* 1.08 (0.93; 1.26) *Gal-3* > *78 ng/ml:* 1.26 (1.08; 1.46)*	*Per 1 Gal-3 SD:* 1.20 (1.10–1.31)* *Gal-3: 56.3–78 ng/ml:* 1.14 (0.93; 1.40) *Gal-3* > *78 ng/ml:* 1.36 (1.11; 1.67)*	–	–	–
2021; Kim	*Per 1 log-Gal-3 unit:* 1.45 (0.93; 2.27)	–	–	–	–
2020; Zhang	–	–	*Per 1 Gal-3 unit:* 1.03 (1.01; 1.06)* *Gal-3* > *30.5 ng/ml:* 1.93 (1.13; 3.28)*	–	–
2020; Wang	–	–	–	*Severe aortic calcification* *Gal-3 tertile 3 vs. 1:* OR: 9.81 (1.76; 54.67)* *Aortic calcification progression* *Gal-3 tertile 3 vs. 1:* OR: 13.29 (3.90; 45.29) *	–
2019; Zhang	–	–	–	*cfPWV: β* = 0.139*	–
2018; Ko	*Per 1 Gal-3 unit:* 1.04 (1.00; 1.08)* *Gal-3* > *30.5 ng/ml:* 2.30 (1.10; 4.90) *	*Per 1 Gal-3 unit:* 1.04 (0.99; 1.10) *Gal-3* > *30.5 ng/ml:* 2.30 (0.80; 6.20)	–	–	–
2016; Hogas	*Gal-3* > *23.7 ng/ml:* 5.4 (2.01; 14.52)*	–	–	–	*Left ventricular ejection fraction: β* = − 0.34*
2016; Obokata	*Per 1 log-Gal-3 unit:* 23.7 (6.45; 86.9)* *Gal-3: 8.1–15.2 ng/ml:* 2.89 (1.04; 8.02) * *Gal-3* > *15.2 ng/ml:* 6.51 (2.52; 16.8)*	–	*Per 1 log-Gal-3 unit:* 50.1 (16.7; 151)* *Gal-3: 8.1–15.2 ng/ml:* 2.13 (0.96; 4.73) *Gal-3* > *15.2 ng/ml:* 7.06 (3.47; 14.40)*	–	–
2015; Gurel	–	–	–	–	*Gal-3 in LVDD vs. non-LVDD:* 23.30 (20.12; 26.87) vs. 14.54 (10.85; 17.65) ng/ml* *E/E΄: r* = 0.645*
2015; Drechsler	*Per 1 Gal-3 unit:* 1.07 (0.98; 1.17)	–	*Per 1 Gal-3 unit:* 1.12 (1.01; 1.24)*	–	–
2015; Yilmaz	–	–	–	–	*Gal-3 in LVH vs. non-LVH:* 11.75 ± 3.56 vs. 5.98 ± 2.75 ng/ml* *Left ventricular mass index: r* = 0.812* *Relative wall thickness: r* = 0.318*

^†^Data represent hazard ratios. Data in parentheses correspond to 95% confidence intervals. Asterisks denote statistical significance (*p-value* < 0.05)

*Gal-3* galectin-3, *SD* standard deviation, *MACE* major adverse cardiovascular events, *baPWV* brachial-ankle pulse wave velocity, *cfPWV* carotid-femoral pulse wave velocity, *LVDD* left ventricle diastolic dysfunction, *LVH* left ventricle hypertrophy

### Cardiovascular mortality

The endpoint of cardiovascular mortality was evaluated in three prospective cohort studies. In one study, higher serum galectin-3 levels were associated with significantly higher cardiovascular mortality risk (HR per standard deviation increase: 1.20, 95% CI: 1.10–1.31), while in two studies, this trend did not reach statistical significance. The exposure–response relationship is schematically presented in Fig. 2b. Higher serum galectin-3 levels were linked to a trend toward increased cardiovascular mortality risk, although a clear dose–response relationship could not be ascertained (*χ*^*2*^: 5.06, *p-value*: 0.079). In comparison to a reference serum galectin-3 value of 10 ng/ml, the risk of cardiovascular mortality was calculated to be significantly higher with galectin-3 levels of 20 ng/ml (HR: 1.58, 95% CI: 1.01–2.47), 30 ng/ml (HR: 2.17, 95% CI: 1.07–4.41) and 40 ng/ml (HR: 2.78, 95% CI: 1.13–6.84).

### Major adverse cardiovascular events

The composite outcome of death or major adverse cardiovascular event was assessed in three prospective cohort studies. When galectin-3 was treated as a continuous variable, its serum levels showed a positive association with the composite outcome risk in all three studies. Two studies introduced cut-offs, analyzing galectin-3 as a binary variable. Specifically, Zhang et al*.*[39] (284 patients) suggested that serum galectin-3 > 30.5 ng/ml is associated with increased risk of death or major adverse cardiovascular event (HR: 1.93, 95% CI: 1.13–3.28), while Obokata et al*.* [45] (423 patients) showed that galectin levels > 15.2 ng/ml were linked to significantly higher risk of the composite endpoint compared to values below 8.1 ng/ml (HR: 7.06, 95% CI: 3.47–14.40).

### Arterial stiffness

The possible association serum galectin-3 with arterial stiffness indices was evaluated in two cross-sectional and one prospective cohort study. Zhang et al. [41] showed that in 311 hemodialysis patients, serum galectin-3 values positively correlated with carotid-femoral pulse wave velocity, while the association remained significant after adjusting for clinical and laboratory covariates (*β*: 0.139, *p-value*: 0.014). Brachial-ankle pulse wave velocity was assessed in a study of 176 patients; although a positive association with galectin-3 levels was observed in the univariate model (*β*: 0.1, *p-value*: 0.03), statistical significance was lost in the fully adjusted multivariate regression model [40]. In addition, serum galectin-3 levels were associated with both abdominal aortic calcification severity (Odds ratio for highest *vs*. lowest tertile: 9.81, 95% CI: 1.76–54.67) and progression (Odds ratio for highest *vs*. lowest tertile: 13.29, 95% CI: 3.90–45.29), suggesting that it may serve as a biomarker with significant predictive efficacy (AUC: 0.71, *p-value* < 0.001) [43].

### Echocardiography

Overall, three studies (two cross-sectional and one prospective cohort) assessed the association of serum galectin-3 with echocardiographic parameters. Hogas et al*.* [38] (88 patients) suggested that higher galectin values were linked to reduced left ventricular ejection fraction (*β*: 0.34, *p-value*: 0.02). Increasing serum galectin-3 values were also associated with left ventricular hypertrophy, positively correlating with left ventricular mass index (*r*: 0.812, *p-value* < 0.001) and relative wall thickness (*r*: 0.318, *p-value* < 0.001) [42]. Additionally, serum galectin-3 levels were found to be significantly higher in hemodialysis patients with left ventricular diastolic dysfunction, presenting a positive correlation with E/E΄ ratio (*r*: 0.645, *p-value* < 0.001) [36].

## Discussion

The present systematic review collected current literature evidence concerning the prognostic role of serum galectin-3 levels in maintenance hemodialysis patients. Dose–response meta-analysis suggested a positive log-linear relationship between serum galectin-3 values and all-cause mortality, while the association with cardiovascular mortality was less clear; although higher galectin-3 levels showed a trend toward higher cardiovascular mortality, the relevant studies were few and statistical significance was not reached. Furthermore, the qualitative synthesis of cross-sectional evidence showed a significant association of serum galectin-3 with arterial stiffness markers, suggesting its potential efficacy as predictor of abdominal aortic calcification severity. Serum galectin-3 correlated also with markers of left ventricular systolic and diastolic dysfunction, also suggesting its possible role in the stratification of heart failure risk among hemodialysis patients.

Serum galectin-3 has been incorporated in combined predictive models, improving their efficacy for cardiovascular risk stratification. Specifically, Miljković et al*.* [31] showed that the combination of serum galectin-3, pentraxin-3, matrix metalloproteinase 9 may be predictive of high cardiovascular risk as assessed by the Framingham risk score (area under the curve: 0.732). In addition, serum galectin-3 has been used in conjunction with *N*-terminal pro-brain natriuretic peptide in a cohort of 173 hemodialysis patients, proposing that the combined model presents significant prognostic value in regards to mortality and cardiovascular events [30]. The combination of serum galectin-3 and pulse wave velocity has been also suggested to improve risk stratification for both cardiovascular and cerebrovascular events [39]. It is important to note that current evidence concerning peritoneal dialysis patients remains limited, although a cross-sectional study has proposed that serum galectin-3 levels are linked to aortic stiffness measured by carotid-femoral pulse wave velocity [33], data regarding its association with hard outcomes are lacking in this population. Limited data are also available for the pediatric population, with a study of 67 children on maintenance hemodialysis showing that serum galectin-3 is associated with left ventricular diastolic dysfunction [35].

Current data suggest that galectin-3 levels may be also linked to adverse clinical outcomes in patients with earlier stages of chronic kidney disease. Specifically, evidence coming from the combined analysis of the Seattle Kidney Study and the Clinical Phenotyping and Resource Biobank Study pointed toward an increased risk of mortality with higher serum galectin-3 levels among patients with pre-dialysis chronic kidney disease [24]. Similarly, the LURIC study indicated that high serum galectin-3 is associated with elevated all-cause and cardiovascular mortality, as well as with sudden cardiac death and death due to infection in chronic kidney disease individuals but not in those with estimated glomerular filtration rate > 90 ml/min/1.73 m^2^ [37]. However, galectin-3 values have shown no association with heart failure or atrial fibrillation incidence [49]. On the other hand, growing evidence suggests that the levels of the biomarker may be linked to progressive renal disease, as reflected by the rate of glomerular filtration rate decline and the progression to end-stage kidney disease [50, 51].

Galectin-3 has been proposed to be implicated in various pathways in the pathophysiology of cardiovascular diseases. In particular, galectin-3 has been suggested to promote increased arterial stiffness [52] and atherosclerosis through its role in inflammation, cell migration and cell–cell interaction [53]. Experimental research has shown that the atherogenic effects of galectin-3 include the recruitment of macrophages in the vascular wall and their differentiation into foam cells, as well as the activation and proliferation of vascular smooth muscle cells leading to extracellular matrix deposition [54]. In addition, serum galectin-3 levels increase in the early phase following acute coronary syndrome since it is involved not only in the development but also in the destabilization of atheromatous plaques [55]. Accumulating evidence points toward the pathogenetic role of galectin-3 in heart failure, as its expression in myofibroblasts may induce the production and deposition of type 1 collagen, promoting cardiac remodeling and myocardial fibrosis [56].

### Strengths and limitations

The present study has several strengths. A systematic literature search has been conducted by screening five databases without date or language restrictions, thus limiting the possibility of any article loss. A dose–response meta-analysis was implemented to delineate the relationship between galectin-3 levels and mortality risk, obtaining thus more robust results compared to a previous conventional meta-analysis in the field [57]. Several important endpoints were also taken into account aiming to provide a comprehensive assessment of galectin-3 prognostic role in hemodialysis patients. On the other hand, the interpretation of the findings is limited by the small number of the included studies per outcome. As a result, the endpoints of arterial stiffness and echocardiography were only qualitatively evaluated since the lack of homogeneous available studies precluded the conduct of quantitative meta-analysis. It should be also noted that these endpoints were mainly assessed by cross-sectional studies, and thus causal associations could not be inferred. In addition, serum galectin-3 was measured using various laboratory kits across different studies, which may lead to heterogeneity of reported absolute values of the biomarker. Cut-off values varied across studies and thus the optimal serum galectin-3 threshold remains to be established. It is important to state that the observational nature of studies along with the lack of appropriate adjustment for covariates may complicate the interpretation of outcomes, as the risk of confounding is not negligible.

### Implications for current clinical practice and future research

The findings of this meta-analysis support the potential utility of serum galectin-3 for risk stratification of patients on maintenance hemodialysis. However, before the biomarker can be incorporated in clinical practice, further research is needed in large scale to validate and extend these outcomes. Specifically, future studies should clarify the relationship between serum galectin-3 and cardiovascular mortality and evaluate additional endpoints, such as the incidence of heart failure, atrial fibrillation and sudden cardiac death. The use of standardized laboratory methods is of great importance in order to introduce cut-off values and provide a realistic estimates of galectin-3 prognostic efficacy. Furthermore, serum galectin-3 should be assessed in conjunction with both traditional risk factors and novel biomarkers, aiming to construct combined models that would enable effective stratification of hemodialysis patients in regards to cardiovascular disease and mortality risk.

## Conclusions

The present systematic review and meta-analysis suggests that high serum galectin-3 levels are associated with increased mortality risk among patients on maintenance hemodialysis. No clear association between serum galectin-3 and cardiovascular mortality could be ascertained. Preliminary cross-sectional data indicate that the biomarker may be linked to arterial stiffness and left ventricular dysfunction. Further research is needed to confirm these findings and elucidate the exact role of serum galectin-3 in clinical practice as a tool for risk stratification of hemodialysis patients.

## Data Availability

All data are available from the corresponding author upon reasonable request.
